# De Chirico and Alice in Wonderland Syndrome: When Neurology Creates Art

**DOI:** 10.3389/fneur.2018.00553

**Published:** 2018-08-13

**Authors:** Domenico Chirchiglia, Pasquale Chirchiglia, Rosa Marotta

**Affiliations:** Department of Medical and Surgical Sciences, Università Degli Studi Magna Græcia di Catanzaro, Catanzaro, Italy

**Keywords:** aura, Todd syndrome, metamorphopsia, headache, dysmetropsia

Giorgio De Chirico (1888-1978), one of the leading exponents of the artistic current of metaphysical painting, was a man of a difficult character: he was often melancholy and sullen, he was also extremely egocentric and so vain as to be considered the most great painter of all time and sign many of his works as “Pictor Optimus.”

De Chirico suffered from a peculiar migraine aura, named “Alice in Wonderland syndrome,” a neurological disorder affecting the perceptual sphere, leading the individual to perceive in an unreal way the size of some parts of his body and outer objects: just like Alice in Lewis Carroll's novel, we find ourselves faced with states of accretion or reduction of their body or of what surrounds us, so who suffers from this disorder suffers a strong distortion of reality, resulting in disorientation and deformation of the senses (1). As described by Todd in 1955, Alice in Wonderland syndrome (AIWS) denotes a series of paroxysmal disorders of the body schema (essential symptoms of AIWS), which may be correlated to phenomena of depersonalization, visual illusions and alterations in the perception of time (2, 3). Giorgio De Chirico did not know that he was suffering from the AIWS, although, in “Memoirs of my Life,” he writes of being suffering from abdominal pain and speaks of “colic saturnine,” referring to Renaissance theory according to which genes are born under the sign of Saturn. Moreover, in a short writing dedicated to Carlo Carrà, De Chirico recounts the experience of headache through a “lucid dream”: “*I sleep. I'm wearing a diver's helmet. The throbbing of my brain splits into many bubbles on the lacquered platform of my seventh ceiling.”*

In order to Chirico's migraine aura as cause of his inspiration, Nicola and Podoll have examined his works as painter and writer, including his “Memoirs,” the autobiographical novels “Hebdomeros” and “Mister Dudron” and his collected essays. Migraine aura symptoms were found by reading clinical descriptions of such disorders, but also observing the paintings and designs from the Migraine Art collection which currently consists of 562 pieces (4).

Migraine symptoms found in Giorgio de Chirico's writings were the following: headache, nausea, photophobia, abdominal pain as autonomic symptoms, scotoma (visual field stain), visual, and gustatory hallucinations, described as spiritual fevers, autokinesis (apparent movement of fixed objects), recurrent dreams, macropsias, micropsias (see objects larger or smaller than normal), telopsias (see objects as very far away), depersonalization syndrome, déjà vu, or jamais vu phenomena. Using the DSM-IV methodology Vanni et al. stated that De Chirico suffered from a personality disorder with narcissistic and paranoid traits and had suffered from somatization disorders in a period between 1909 and 1918 (5). Psychiatric disorders have long been studied. Personality disorders, anxiety, depression, dissociative disorders do not manifest with warning signs such as auras. They are structured disorders, with well-defined characteristics. For example, depression can have, as symptoms, crying crises, changes in sleep, loss of pleasure. Psychosis can manifest hallucinations, but these are symptoms and not auras (4). Blanke about some symptoms that De Chirico described, affirms these are encountered in patients with temporal lobe epilepsy, suggesting, they rather have related to temporal lobe epilepsy than migraine (6). Temporal lobe epilepsy includes physical and psychic symptoms. More than 90% of patients with temporal lobe epilepsy report an epigastric aura. Other autonomic symptoms, psychic symptoms, and certain sensory phenomena (such as olfactory) also occur. Headache is not reported. The duration of complex partial seizures is generally >1 min, while somatosensory and visual auras are highly unlikely with temporal lobe epilepsy (7). There is a long discussion about the origin of De Chirico's disorders: epilepsy, psychiatric disorders or migraine? Although some of the symptoms may be related to temporal lobe epilepsy or some forms of psychosis (déjà vu phenomenon), or epigastric pain crises, visual aura as in migraine, it is also true that from the painter's autobiography as from the various biographies, the reported symptoms, especially for durability and morphology (metamorphopsias) are more compatible with the aura of AIWS (4, 7). In short, the key to reading De Chirico's disorder is in migraine with visual-somal-sensory aura. typical of AIWS. From the pictorial works it is deduced that the aura of the artist was different from the epileptic aura, especially for the longest duration and for the alterations of things, figures, people, and more, characteristics of AIWS (7). The confirmation is given by the pictorial works: the telopsia-macropsia in Piazza d' Italia, 1913, or the metamorphopsia in the series of mannequins. In Piazza d' Italia there is a long perspective where some people are very small compared to tall colonnaded buildings, while the mannequins have an oval shaped head, without eyes, ears, mouth, representing a visual depersonalization.

De Chirico's autobiography and essays as well as his first metaphysical painting (The Enigma of an Autumn Afternoon, 1909) are the proof that migraine visual aura phenomena, associated to paramnesias (jamais and déjà vu) could be interpreted as the start of the painter's creative process (4). The link between migraine and AIWS, hypothesized by Todd (1), is the most reported in the literature and is the line mainly considered in the framing of the syndrome. The information available to date is supported by data derived from the use of techniques of transcranial magnetic stimulation and neuroimaging. Hamed, referring to a case of coincidence between migraine and AIWS, states the implication of the occipital lobe and the parietal lobe (8). This data is confirmed by the study of Brumm et al. who first recorded the cerebral activity of a patient with the syndrome, during an attack of micropsia. Using functional magnetic resonance imaging (fMRI), which allows the activity of cortical areas to be described on the basis of blood flow detection, the authors have detected abnormal activation of the occipital lobe, in the primary regions and in the extrastriatal parts of visual cortical areas, and of the parietal lobe (9). The results are also in agreement with what was claimed by Cau on the possibility of the manifestation of perceptual alterations, such as those present in AIWS, in the case of temporal-occipital and parieto-occipito-temporal lesions, emphasizing the role of these areas in the formation of perceptions (10).

## The man and the artist, the double

The concept of the double has attracted the attention of scientists. A documentary film by Daniel Müller, “The Second Body” (2003) includes a sequence about Giorgio de Chirico's double as represented in his self-portrait (with shadow) from 1920. The white shape of a life-sized human body with the characteristic profile of De Chirico's head is pictured back to the artist's physical body, represented in full color and details. The white human-shaped figure is located behind the artist's head, which excludes an interpretation in terms of view of own mirror image (autoscopy) or seeing one's double, but rather indicates an out-out-body experience(OBE) with the somesthetic sensation of a double body with an extracampine localization behind the physical body (8). The apparent union of the own physical body and his duplicate body suggests that De Chirico has pictured the process of either separation or return of the parasomatic body from or to the physical body, respectively. Out-of-body experience, which may occur as a migraine aura symptom, complies with interpretation of the artist's self-portrait with shadow. Thus, Wieland Schmied wrote: “Behind the artist in his dark jacket a bright, almost white shadow turns away. It is as if his alter ego detached itself from him, as if a part of his self-left him” (11). Fagiolo dell'Arco gave the following interpretation: “The most surprising peculiarity consists of the white shadow which detaches itself from his body and looks outwards, transforming into an animistic double” (12). In essay “Metaphysical art and occult sciences,” the painter has referred to “the fact of considering the possibility of existence of immaterial forms, of imagining a double of ours…made up of fluids and of incorporeal substances”. “Metaphysical painting” means an art that uses the technical tools typical of painting (perspective, chiaroscuro, color) to represent something that goes beyond sensory experience, leaving room for dreams and visions fruit of the unconscious. In the metaphysical painting also the places, however realistic, assume a dreamlike value because of a distorted perspective, of elements apparently out of place (statues, mannequins) and of unnatural colors (13). Suggestive elements of De Chirico's metaphysical works are the immense squares without human presence where bizarre elements emerge such as mannequins, marble busts, and classic columns. These paintings reveal a sense of loneliness and restlessness, as if one lived in a strange dream (14). It is a particular disorder that leads those who suffer to have a visual perception bizarre, just as happened to Alice after entering the lair of the Rabbit: parts of the body that seem to become enormous, rooms of the house that shrink, Toys that take the size of buses (10).

This particular syndrome is a real neurological disorder, which suddenly appears in patients who suffer and generally disappears after various periods, which can last from months to years.

## Conclusions

Giorgio De Chirico suffered unquestionably from AIWS. Techniques of neuroimaging offered a valid apport to knowledge of anatomical and functional aspects about the syndrome. Generally parietooccipital brain lobes and particularly temporoparietal-occipital carrefour (TPO-C) are supposed as regions for developing many of AIWS symptoms. AIWS symptoms depend on an alteration of TPO-C where visual-spatial and somatosensory information are integrated. Alterations in these brain regions may cause dysmetropsias and disorders of body schema. Clinical symptoms, together with neuroimaging, confirm what has been said. De Chirico's aura was not an epileptic aura, nor a psychiatric aura. It was a visual -somesthetic aura, in the context of a migraine. These considerations are reflected in the data of the literature, in the autobiographies, biographies, and various writings, concerning the artist. It is really extraordinary to think that a neurological disorder can generate such wonders. Therefore, it is the case to say “when neurology creates art.”

## Author contributions

PC provided to prepare manuscript. RM provided to elaborate manuscript. DC provided to approve manuscript.

### Conflict of interest statement

The authors declare that the research was conducted in the absence of any commercial or financial relationships that could be construed as a potential conflict of interest.
